# The Medical Society of the State of New York

**Published:** 1890-03

**Authors:** 


BUFFALO MEDICAL AND SURGICAL JOURNAL
A MONTHLY REVIEW OF MEDICINE AND SURGERY.

*EDITORS:*
THOS. LOTHROP, M. D. - - - - W. W. POTTER, M. D.
All communications, whether of a literary or business character, should be addressed to the editors: 284 Franklin Street, Buffalo, N. Y.

### Editorial.

### THE MEDICAL SOCIETY OF THE STATE OF NEW YORK.

The Eighty-fourth Annual meeting of this ancient and honorable medical organization, held in Albany, Tuesday, Wednesday and Thursday, February 4, 5, and 6, 1890, was well attended, and the work accomplished was in every way praiseworthy.

The Committee on Revision of the By-Laws, through its Chairman, Dr. Henry Flood, of Elmira, presented a report that was adopted, with the exception of the clause relating to the Nominating Committee. We think this Committee of Revision, and especially its Chairman, is entitled to the thanks of the Society for the able and judicious manner in which it has performed a laborious and time-consuming duty. It was a work that must be done, and it is difficult to see how it could have been done better. It necessarily took time to act upon this report, which must be done in open session, and we fail to see how any fair criticism can be offered upon the fact that this and other executive business consumed two hours on the morning of the first day. If the genial editor of the *Medical Record* would attend the meetings and take active part in the business thereof, perhaps he might find less occasion to criticise those who are obliged to "manage the Society." He is a member of an important committee, and has been for several years, but we have failed to observe his attendance upon the sessions for some time past.

The scientific papers read at this meeting were of a high order of merit, and the discussions were able and instructive. To President Lewis, and his able lieutenants of the Business Committee, all praise must be accorded for such substantial results. To arrange in systematic order, and bring forward in tangible shape for consideration, such a vast amount of material as is yearly offered and disposed of in this Society, is no slight task; and when the work is well done, it is becoming that a word of approbation for those who perform the laborious and gratuitous service should be given.

The Society reaffirmed its interest, maintained these many years, in the proposed State Board of Medical Examiners. The propriety of separating the teaching and licensing bodies for the practice of medicine, has gone past the stage of argument—it is an admitted necessity. The only question that remains is as to the method of accomplishing this important reform. This Society is of the opinion that Senate bill 115, introduced by Senator Stadler, and now pending, is well calculated to do equal and exact justice to all interests involved. A committee of fifteen was appointed to appear before the Senate Committee on Public Health, and urge the passage of this bill. A hearing has already been accorded the Committee, the arguments have been presented, and we await the result, not doubting that the Legislature of the State of New York will show its readiness to take such action, as will array the Empire State well in the front rank with her sister commonwealths that have already placed similar laws upon their statute books. To doubt this would be to challenge the intelligence and progressiveness of the men who have in custody all that is dear to the citizens, whose servants they are.

---

The banquet held at the Delavan House on the second night of the meeting was of more than ordinary interest. The speeches were unusually witty and entertaining, the presence of the Hon. St. Clair McKelway, of Brooklyn, a familiar face to the old banqueters, adding not a little to the enjoyment of the evening. But the climax of the feast was reached when Drs. Jacobi and Roosa made their stirring appeals in behalf of the Medical Examiners' bill. Inspired in part, no doubt, by the presence of Senator Fassett, whose wit and eloquence had already charmed the company beyond measure, and by that of other members of the Legislature who graced the occasion, Dr. St. John Roosa fairly carried the assemblage to its feet in round after round of applause with the lofty outburst of fervid eloquence of his peroration, in which he set forth the importance of the measure in question, and its bearings upon the future welfare and prosperity of the people. It was a scene not to be described in words, and never to be forgotten by those who were so fortunate as to witness it.

---

The officers elected for the ensuing year were: *President*, Dr. William Warren Potter, of Buffalo; *Vice-President*, Dr. Lewis S. Pilcher, of Brooklyn; *Secretary*, Dr. Frederick C. Curtis, of Albany; *Treasurer*, Dr. Charles H. Porter, of Albany. The usual standing committees were filled for the most part by electing the former incumbents; a large number of delegates to other societies were appointed, and a number of honorary members were chosen.

